# Baicalin, a natural compound, promotes regulatory T cell differentiation

**DOI:** 10.1186/1472-6882-12-64

**Published:** 2012-05-16

**Authors:** Ji Yang, Xue Yang, Ming Li

**Affiliations:** 1 Department of Dermatology, Zhongshan Hospital, Fudan University, Shanghai 200032, China; 2 Division of Rheumatology, Huashan Hospital, Fudan University, Shanghai China

**Keywords:** Baicalin, Foxp3, Treg cells

## Abstract

**Background:**

CD4^+^CD25^+^Foxp3^+^ regulatory T (T_reg_) cells inhibit autoimmunity and protect against tissue injury. The development of these T_reg_ cells is controlled by the regulator protein Foxp3, which can be enhanced by the *in vitro* activation of Foxp3 in the presence of transforming growth factor-beta. However, little is known about alternative methods, such as the use of natural products, for controlling Foxp3-mediated T_reg_ cell differentiation.

**Method:**

HEK 293 T cells were transfected with Foxp3 expression plasmid, and then treated with different compounds, Foxp3 mRNA expression was determined by real-time RT-PCR. CD4^+^CD25^-^T cells were stimulated with Baicalin, Foxp3 protein expression were analyzed by flow cytometry and confocal microscopy, the regulatory function of T cells stimulated with Baicalin was detected by the carboxyfluorescien succinimidyl ester.

**Results:**

We demonstrated that Baicalin, a compound isolated from the Chinese herb Huangqin, induced Foxp3 protein expression in cultured T cells, promoted T_reg_ cell differentiation and regulatory activity. Our data also indicated that Baicalin restored Foxp3 expression following its initial interleukin-6-mediated inhibition and induced Foxp3 expression *in vitro*.

**Conclusions:**

These data suggest that Baicalin may promote T_reg_ cell differentiation and regulatory activity and may serve as a promising natural immunosuppressive compound for treating autoimmune inflammatory diseases.

## Background

CD4^+^CD25^+^Foxp3^+^ regulatory T (Treg) cells are essential for maintaining self-tolerance [1,2] and play key roles in regulating immune system homeostasis [2]. Increasing evidence suggests that T_reg_ cells are capable of inhibiting the function of Th1, Th2, Th17, and other effector cells; inhibiting inflammation and preventing autoimmunity [3,4]; therefore, the loss or dysfunction of T_reg_ cells often leads to autoimmune diseases such as systemic lupus erythematosus [5] , type I diabetes and inflammatory bowel disease [2,6]. The development and functions of T_reg_ cells are driven by the forkhead/winged-helix transcription factor Foxp3 [7-9], whose important role in T_reg_ cell activity was previously demonstrated when mice expression mutant Foxp3 exhibited impaired T_reg_ cell activity and succumbed to a fatal lymphoproliferative disorder, which was then reversed by the transgenic expression of wild-type Foxp3 in these animals [10].

T_reg_ cells typically develop and proliferate in the thymus upon contact with self-antigen and are then transported to the peripheral immune system [2]. Several factors affecting T_reg_ cell homeostasis have been defined: for example, T_reg_ cell activity is interleukin (IL)-2-dependent [11]. In addition, T_reg_ cell activity can be induced in the peripheral immune system by the conversion of naïve CD4^+^Foxp3^-^ T cells into Foxp3^+^ T cells by transforming growth factor-beta (TGF-β) [4], which also maintains Foxp3 expression and can regulate the functions of T_reg_ cells [12]. Foxp3 is essential for the development and function of CD4^+^CD25^+^ regulatory T cells [13], induction of the transcription factor Foxp3 can converse CD4^+^CD25^-^ naïve T cells to CD4^+^CD25^+^ regulatory T cells [14]. In addition, Foxp3 solidifies T_reg_ cell lineage and amplifies the pre-established T_reg_ cells [7]. The effect of Foxp3 is to suppress the activation of target genes on T cell stimulation, as well as inhibit the production of interleukin (IL)-2, therefore Foxp3 is crucial for the normal function of T_reg_ cells [15]. Thus induction of Foxp3 expression and promotion of T_reg_ cells differentiation might be a promising therapeutic method for the treatment of autoimmunity diseases.

Recently, the vitamin A metabolite all-trans retinoic acid was shown to enhance TGF-β-mediated induction of Foxp3 [16-18]. In traditional Chinese medicine, some herbs are used in various anti-inflammatory applications [19-22], such as the treatment of autoimmune diseases, by suppressing effector T-cell reactions [23]; however, little is known about the role of natural compounds in controlling the differentiation and functions of T_reg_ cells. Since T_reg_ cells regulate the functions of effector T cells, we hypothesized that some therapeutic herbs may suppress inflammation by promoting Foxp3-mediated T_reg_ cell differentiation and thus controlling effector T cell activities. To support this, previous study showed that berberine, an isoquinoline alkaloid derived from plants, inhibited differentiation of Th17 cells and, to a lesser degree, Th1 cells, whereas it had no effect on the relative number of CD4^+^Foxp3^+^ regulatory T cells in the experimental autoimmune encephalomyelitis mice [19]. Artemisinin analog SM934 had therapeutic effects in lupus-prone female MRL*/lpr* mice by inhibiting both Th1 cell and Th17 cell responses, and elevated the percentage of Treg cells ex vivo, but not Treg cells in vitro[22].

In this study, we selected herbs for the development of Treg cells, two criteria are considered: the herb must be safe for patient use, and the chemical structures of the active ingredients of the herb must already be described. We demonstrated that Baicalin, a flavonoid compound originally isolated from the Chinese herb Huangqin (*Scutellaria baicalensis* Georgi), up-regulated Foxp3 mRNA expression in HEK 293 T cells and promoted T_reg_ cell differentiation. Furthermore, we showed that Baicalin could restore Foxp3 expression following its initial IL-6-mediated inhibition, Baicalin could also directly induce Foxp3 expression, promote T_reg_ cell differentiation and regulatory activity. Our findings indicate that Baicalin may be a promising natural immunosuppressive compound for treating inflammatory diseases.

## Methods

### Plasmids, cell lines, and transfection for Foxp3 mRNA expression studies

The Foxp3-IRES-GFP expression plasmid (pZIGF) and control plasmid were kindly provided by Wang Shengjun. HEK 293 T cells (Chinese Academy of Sciences, Shanghai, China) were maintained in Dulbecco’s modified Eagle’s medium (Hyclone, Logan, UT) and transiently transfected with pZIGF using Lipofectamine (Invitrogen, San Diego, CA). At 24 h post-transfection, HEK 293 T cells were treated with different compounds (20 μM Baicalin, 10 μM Paeoniflorin, 0.5 ng/ml Triptolide, 1 μM Artemerher, 5 μM Cryptotanshinone, 30nM Retinoic acid, 10 μM Sinomenine, or 10 μM Paeonol; All compounds are from National Institute for the Control of Pharmaceutical and Biological Products, Beijing, China) for another 24 h, and Foxp3 mRNA expression levels from cells in each group were determined by real-time reverse transcription-polymerase chain reaction (RT-PCR). For some experiments, HEK 293 T cells were treated with different doses of Baicalin or incubated with a constant dose of Baicalin (20 μM) for various treatment times, and Foxp3 mRNA expression was analyzed by real-time RT-PCR. Where indicated, cultures were supplemented with 5 ng/ml TGF-β, 20 ng/ml IL-6 (PeproTech, Rocky Hill, NJ), 20 μM Baicalin, or DMSO vehicle control, and Foxp3 mRNA expression was determined by RT-PCR.

### RNA isolation and real-time RT-PCR

Total RNA from transfected HEK 293 T cells was prepared with the use of the Trizol reagent according to the manufacturer’s protocol (Invitrogen). cDNA was synthesized with a first-strand cDNA synthesis kit and oligo (dT) primers (Fermentas, Hanover, MD), and gene expression was examined with a Bio-Rad iCycler Optical System (Bio-Rad, Richmond, CA) using a SYBR green real-time PCR Master Mix (Toyobo, Osaka, Japan). The 2-ΔΔCt method was used to normalize transcription levels to human 18S or Mus β-actin and to calculate fold-changes in expression levels relative to controls. The primer pairs could be seen in Table 1.

**Table 1:** The following primer pairs were used

**Gene**	**Forward (5'-3')**	**Reverse (5'-3')**
Mus β-actin	GACGGCCAGGTCATCACTATTG	AGGAAGGCTGGAAAAGAGCC
Mus Foxp3 (239 T cell)	CTACCCACTGCTGGCAAATG	GCCTTGCCTTTCTCATCCA
Mus Foxp3 (T cell)	CCCAGGAAAGACAGCAACCTT	TTCCACAACAAGGCCACTTG
Human 18S	GCCCGAAGCGTTTACTTTGA	TCCATTATTCCTAGCTGCGGTATC
Human IL-6Rα	CATTGCCATTGTTCTGAGGTTC	AGTAGTCTGTATTGCTGAGGTTC

### Mice, CD4^+^ T-cell isolation, and culture conditions

Wild-type C57BL/6 (B6) mice were purchased from the Shanghai Laboratory Animal Center (Chinese Academy of Sciences). The animal protocol was approved by the Institutional Animal Use Committee of the Shanghai Institutes for Biological Sciences. All mice were maintained under pathogen-free conditions. Naïve CD4^+^CD25^-^ T (FITC-conjugated anti-CD4 and PE-conjugated anti-CD25, eBioscience, San Diego, CA) cells from spleen of B6 mice were isolated by fluorescence-activated cell sorting (FACS). T cells were stimulated with 2 μg/ml plate-bound anti-CD3 and 2 μg/ml soluble anti-CD28 (eBioscience) for 3 days. Where indicated, cultured T cells were treated with different doses of Baicalin, and a DMSO-only control was used in some experiments. For some experiments, CD4^+^CD25^-^ T cells were labeled by carboxyfluorescien succinimidyl ester (CFSE, Invitrogen), and co-cultured with CD4^+^CD25^+^T cells in the presence of 20 μM Baicalin for 5 days, the proliferation of T cells was analyzed by flow cytometry.

### Flow cytometry analysis and confocal microscopy

Cells obtained from in vitro cultures were first stained with FITC-conjugated anti-CD4, PE-conjugated anti-CD25 (eBioscience). For Foxp3 staining, cells were treated with PE-Cy5-conjugated anti-Foxp3 according to the manufacturer’s protocol (eBioscience). After staining, cells were analyzed in a FACS-Calibur flow cytometer (BD-Bioscience) using FlowJo software (Tree Star, San Carlos, CA). Cells were then visualized with a Leica TCS SP2 confocal microscope (Leica, Cambridge, UK), and fluorescence intensity data were measured using LCS Lite software (Leica). Five independent cells were selected randomly from each sample for analysis.

### Statistical analysis

Quantitative data were expressed as means ± standard deviation (SD). The statistical significance was determined by analysis of variance followed by a Bonferroni *post-hoc* test for multiple comparisons or the Student’s t-test. All *p* values ≤ 0.05 were considered significant.

## Results

### Screening of compounds up-regulating Foxp3 expression

**Figure 1:**
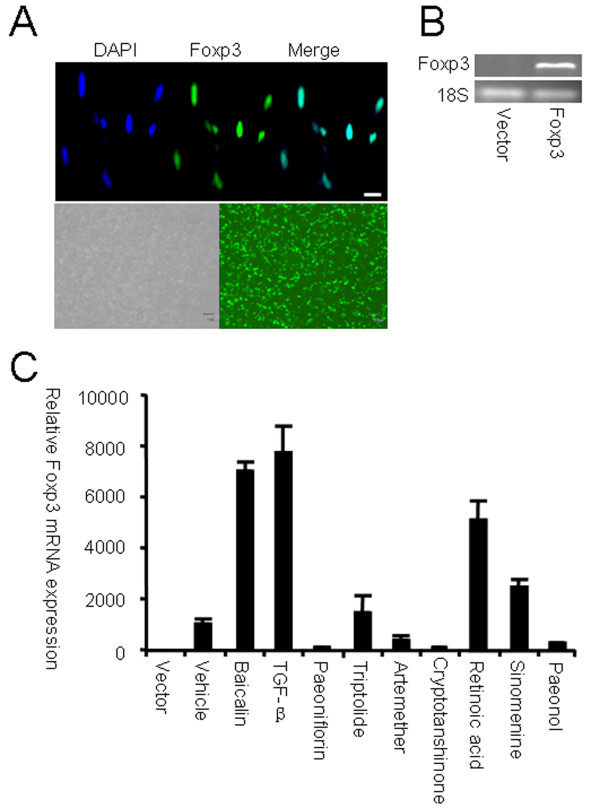
**Screening of compounds that up-regulate Foxp3 expression.** HEK 293 T cells were transfected with Foxp3 expression plasmids. ( **A**) Nuclear expression of Foxp3 in HEK 293 T cells was examined by immunofluorescence microscopy. Scale bar, 10 μm. ( **B**) Foxp3 mRNA expression in transfected HEK 293 T cells was determined by RT-PCR. ( **C**) Twenty-four hours after transfection, cells were then treated with different compounds for another 24 h. Foxp3 mRNA expression was analyzed by real-time RT-PCR. Data are expressed as mean ± SD, and compared with vector control (expression level of vector control was set as 1.0). Shown are results from three independent experiments.

To screen for natural compounds that up-regulate nuclear transcription factor Foxp3 expression in T cells, HEK 293 T cells were transiently transfected with the Foxp3-GFP expression plasmid pZIGF. Subsequent immunofluorescence microscopy showed the nuclear localization of Foxp3 in the cells (Figure 1A), which allowed us to accurately identify compounds that affect the gene expression of Foxp3. Real-time RT-PCR confirmed that Foxp3 mRNA expression levels were higher in pZIGF-transfected HEK 293 T cells when compared with vector-only control cells (Figure 1B). To identify novel compounds that up-regulate Foxp3 expression, the transfected HEK 293 T cells were then treated with different anti-inflammatory compounds isolated from Chinese herbs at concentrations that would not adversely affect cell proliferation (data not shown). Among the different compounds tested, Baicalin (7-glucuronic acid, 5, 6-dihydroxyflavone, molecular weight = 446.36) induced the highest expression of Foxp3 mRNA (Figure 1C). We confirmed this finding by stimulating HEK 293 T cells with combinations of TGF-β and different herbal compounds and observed a synergistic effect of TGF-β and Baicalin with respect to Foxp3 mRNA expression (data not shown).

### Baicalin up-regulates Foxp3 expression in HEK 293 T cells

**Figure 2:**
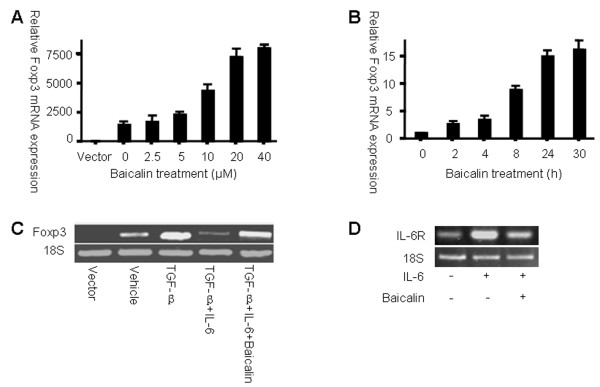
**Baicalin up-regulates Foxp3 expression in HEK 293 T cells.** Foxp3 mRNA levels in transfected HEK 293 T cells are shown ( **A**) Twenty-four hours after transfection, cells were treated with Baicalin for another 24 h. The dose-dependent induction of Foxp3 mRNA expression is shown. Date are expressed as mean ± SD and compared with the vector control (expression level of the vector control was set as 1.0). ( **B**) After transfection with Foxp3 expression plasmids, a second group of cells was treated with 20 μM Baicalin. The time-dependent induction of Foxp3 mRNA expression is shown. Data are expressed as mean ± SD (expression level at 0 h was set as 1.0). ( **C**) Transfected HEK 293 T cells were treated with indicated cytokines in the presence or absence of Baicalin for another 24 h. Foxp3 mRNA was examined by RT-PCR. ( **D**) HEK 293 T cells were treated with IL-6 in the presence or absence of Baicalin for 24 h, and IL-6R mRNA levels were examined by RT-PCR.

After transfection with pZIGF, HEK 293 T cells were treated with Baicalin, which resulted in both dose- and time-dependent increases in the expression of Foxp3 mRNA (Figure 2A and B). Because TGF-β can induce the expression of Foxp3 and inflammatory cytokines such as IL-6 may inhibit its expression [24], we studied Baicalin’s effects on Foxp3 expression in the presence of TGF-β and IL-6. As expected, the addition of TGF-β and IL-6 to transfected cells inhibited Foxp3 mRNA expression, whereas the subsequent addition of Baicalin could restore Foxp3 expression (Figure 2C) by countering the activity of IL-6. Furthermore, our data showed that treating transfected HEK 293 T cells with IL-6 could induce the expression of IL-6R mRNA and that treatment with Baicalin could inhibit IL-6 receptor mRNA expression (Figure 2D).

### Baicalin promotes Foxp3 expression in CD4^+^CD25^-^ T cells

**Figure 3:**
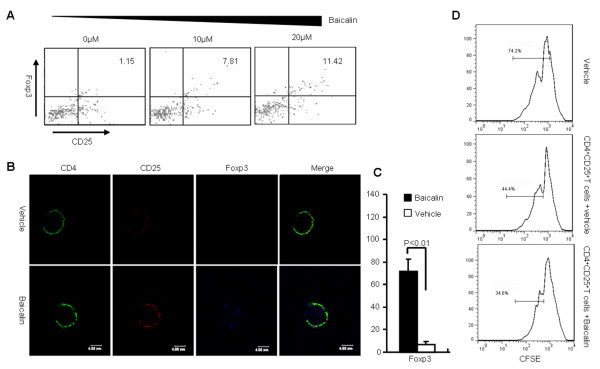
**Baicalin up-regulates Foxp3 expression in CD4**^**+**^**CD25**^**-**^**T cells.** ( **A**) FACS-sorted CD4^+^CD25^-^ T cells from B6 mice were stimulated with indicated doses of Baicalin for 3 days. Foxp3 expression in the gate of CD4^+^ T cells was determined by flow cytometry. (**B**) CD4^+^CD25^-^ T cells from B6 mice were cultured in the presence or absence of Baicalin for 3 days. Using confocal microscopy, nuclear Foxp3 expression was detected in the CD4^+^ T cells. The scale bar represents 4 μm. (**C**) Five independent cells were selected randomly from each sample, and shown are results from three independent experiments. ( **D**) CFSE labeled CD4^+^CD25^-^ T cells were co-cultured with CD4^+^CD25^+^ T cells at a 2:1 ration in the presence or absence of Baicalin for 5 days, the proliferation of cells was determined by flow cytometry.

To determine the effects of Baicalin on endogenous Foxp3 expression, CD4^+^CD25^-^ T cells from wild-type B6 mice were sorted by FACS and activated with anti-CD3 and anti-CD28 antibodies in the presence of Baicalin. Our data showed that Baicalin could induce the differentiation of these T_reg_ cells in a dose-dependent manner (Figure 3A). Confocal microscopy of the activated cells revealed nuclear expression levels of Foxp3 protein in CD4^+^CD25^-^T cells after a 3-day treatment with Baicalin (Figure 3B, C). Further study showed that in the presence of Baicalin CD4^+^CD25^+^T cells have stronger inhibition on the proliferation of effector T cells (Figure 3D). These data demonstrate that Baicalin could promote nuclear Foxp3 expression, induce CD4^+^CD25^+^ Foxp3^+^ T_reg_ cell differention and strengthen the regulatory function.

## Discussion

Baicalin, an active ingredient originally isolated from the root of the Chinese herb Huangqin (*Scutellaria* baicalensis Georgi), has been used as an anti-inflammatory drug in traditional Chinese medicine practices [25,26]. Previous studies have investigated Baicalin’s mechanisms of action, revealing that Baicalin could suppress the production of inflammatory mediators such as IL-6 [25], bind to a variety of chemokines to limit their biological functions [27], and prevent leukocyte adhesion [28]. In addition, we have previously shown that Baicalin inhibited Th17 cell differentiation both *in vitro* and *in vivo*, Baicalin could restore IL-6-mediated inhibition of Foxp3 [29]. But whether Baicalin could promote Foxp3 expression in the absence of TGF-β is not clear, whether T_reg_ cells generated in the presence of Baicalin maintain regulatory activity is not known.

In the current study, we found that Baicalin up-regulated both exogenous and endogenous Foxp3 expression and subsequently counteracted IL-6 mediated inhibition of Foxp3 expression, which confirmed previous findings that Foxp3 expression was restored after its initial suppression of specific pro-inflammatory cytokines [24]. TGF-β induces the differentiation of T_reg_ cells, whereas IL-6 inhibits TGF-β-induced generation of T_reg_ cells [17], and IL-6 blockade by treatment with an anti-IL-6R monoclonal antibody could promote T_reg_ cell differentiation [30]. Our data showed that Baicalin could inhibit IL-6R mRNA expression, which implied that Baicalin could restore IL-6-IL-6R-mediated inhibition of Foxp3 expression during T_reg_ cell differentiation. We further determined that Baicalin could directly up-regulate the expression of Foxp3 in transfected HEK 293 T cells and cultured T lymphocytes in the absence of TGF-β. In addition, we confirmed that Baicalin induced the nuclear expression of Foxp3 and promote T_reg_ cell differentiation *in vitro.* T cells stimulated with Baicalin showed more powerful inhibition on the proliferation of effector T cells, which indicated the expression of Foxp3 is correlated to the T_reg_ cell activity. Although our data showed that Baicalin could directly promote Foxp3 expression in T cells, further investigations should explore the possible mechanisms, such as epigenetic regulation.

Autoimmune responses and homeostasis are maintained by a fine balance between effector T and T_reg_ cell activities. In patients with autoimmune diseases, the proliferation of effector T cells is directly related to the depletion and/or dysfunction of T_reg_ cells [31]. During an immune response, feedback regulators initiate and activate effector cell activities, so the exclusive inhibition of specific effector T cells may not effectively control the response. Therefore, for the treatment of autoimmune diseases, therapeutic agents that can regulate the relationship between effector T cells and T_reg_ cells rather than those that only regulate effector T cells are of great clinical interest [31]. Our data showed that Baicalin could promote T_reg_ cell differentiation and up-regulate the regulatory function of T_reg_ cells *in vitro*, which imply that Baicalin might be used for the treatment of autoimmune diseases. Whereas further study should be done to elevate the role of Baicalin on the differentiation of Treg cells *in vivo*.

## Conclusions

In this study, we demonstrated that Baicalin could enhance the expression of Foxp3, potentially promoting T_reg_ cell differentiation and activity *in vitro*. Specifically, the Foxp3-mediated induction of T_reg_ cells may control autoimmune diseases by eliminating inflammation caused by effector T cells; thus Baicalin might serve as a promising natural immunosuppressive compound for the treatment of autoimmune diseases.

## Competing interests

The authors declare that they have no competing interests.

## Authors’ contributions

Ji Yang and Xue Yang carried out the cell culture, transfection and drafted the manuscript. Ji Yang and Xue Yang carried out molecular biology studies and immunoassays. Ji Yang, Xue Yang, and Ming Li participated in the design of the study and performed the statistical analysis. Ji Yang, Xue Yang, and Ming Li conceived of the study, and participated in its design and coordination. All authors read and approved the final manuscript.

## Pre-publication history

The pre-publication history for this paper can be accessed here:

http://www.biomedcentral.com/1472-6882/12/64/prepub
